# How Does Emergency Department Crowding Affect Medical Student Test Scores and Clerkship Evaluations?

**DOI:** 10.5811/westjem.2015.10.27242

**Published:** 2015-11-12

**Authors:** Grant Wei, Rajiv Arya, Z. Trevor Ritz, Albert S. He, Pamela A. Ohman-Strickland, Jonathan V. McCoy

**Affiliations:** * Rutgers Robert Wood Johnson Medical School, Department of Emergency Medicine, New Brunswick, New Jersey; † Rutgers School of Public Health, Department of Biostatistics, New Brunswick, New Jersey

## Abstract

**Introduction:**

The effect of emergency department (ED) crowding has been recognized as a concern for more than 20 years; its effect on productivity, medical errors, and patient satisfaction has been studied extensively. Little research has reviewed the effect of ED crowding on medical education. Prior studies that have considered this effect have shown no correlation between ED crowding and resident perception of quality of medical education.

**Objective:**

To determine whether ED crowding, as measured by the National ED Overcrowding Scale (NEDOCS) score, has a quantifiable effect on medical student objective and subjective experiences during emergency medicine (EM) clerkship rotations.

**Methods:**

We collected end-of-rotation examinations and medical student evaluations for 21 EM rotation blocks between July 2010 and May 2012, with a total of 211 students. NEDOCS scores were calculated for each corresponding period. Weighted regression analyses examined the correlation between components of the medical student evaluation, student test scores, and the NEDOCS score for each period.

**Results:**

When all 21 rotations are included in the analysis, NEDOCS scores showed a negative correlation with medical student tests scores (regression coefficient= −0.16, p=0.04) and three elements of the rotation evaluation (attending teaching, communication, and systems-based practice; p<0.05). We excluded an outlying NEDOCS score from the analysis and obtained similar results. When the data were controlled for effect of month of the year, only student test score remained significantly correlated with NEDOCS score (p=0.011). No part of the medical student rotation evaluation attained significant correlation with the NEDOCS score (p≥0.34 in all cases).

**Conclusion:**

ED overcrowding does demonstrate a small but negative association with medical student performance on end-of-rotation examinations. Additional studies are recommended to further evaluate this effect.

## INTRODUCTION

Emergency department (ED) crowding has been described in emergency medicine (EM) literature as a concern for over 20 years.^1^ Previous reports have noted crowding as a risk factor for patients leaving without being seen, increased inpatient mortality, increased frequency of medical errors, and increased length of stay for all patients.^2^–^6^ Solutions have been proposed with the implementation of surge protocols and improvement of both ED workflow and downstream factors.^7^–^8^ In the academic medical setting, an additional concern is the impact of ED crowding on resident and medical student education.

Relatively few studies exist in the medical literature assessing the effect of ED crowding on the educational outcomes of residents and medical students. Investigations that have been completed do not generally find significant associations between crowding and educational quality. Pines et al. found no association between crowding metrics and resident/medical student assessment of attending physician teaching.^9^ Mahler did find that during overcrowded periods, residents saw fewer patients and performed fewer procedures; however, resident physicians judged the quality of their education as unaffected.^10^ Perceptions of crowding did not correlate with the perception of educational quality in another study.^11^

The reason for these outcomes may not be immediately obvious, as an intuitive understanding of ED crowding suggests that limitations on space, resources, and attending physician time in periods of crowding would negatively impact secondary goals such as education. It has been speculated that attending physicians may simply prioritize education regardless of crowding status or that crowding status itself may not necessarily impose additional workload on the ED attending physician.^9^ Alternatively, less severe ED crowding may improve resident education by increasing the opportunity for residents to see higher volumes and higher acuity patients.^12^ Evidentiary support for these ideas is limited.

Several measures of ED crowding have been described previously in the literature. The ED Work Index (EDWIN) identifies ED crowding according to a conceptual formula, with good accordance with physician and nurse impressions of crowding.^13^ The National ED Overcrowding Scale (NEDOCS) uses five operational variables in a logistic regression model to identify periods of crowding.^14^ Agreement between the models is high and both have good discrimination for prediction of ED crowding.^15^

Given the lack of data in this area, we sought to determine whether ED crowding, as measured by the NEDOCS score, has a quantifiable effect on medical student objective and subjective experiences during the EM clerkship rotations.

## METHODS

### Study Setting and Population

Our setting is an urban Level It rauma center with ED residents, a required fourth-year medical student clerkship, a 19-member academic faculty, and an approximate annual volume of 70,000 adult patients. The facility contains a separate dedicated pediatric ED as well; data for this study pertained exclusively to the adult ED. The medical student rotation at this facility is scheduled in four-week blocks occurring between July and April with a single combined eight-week block from December-January. Between four and 21 students rotate through the adult ED at a time, completing nine-hour shifts that fall predominately between 9 a.m. and 11 p.m. Medical students are paired up individually with an attending for their shifts, and while there are often other learners (i.e., residents) with the attending there is never more than one clerkship student with an attending.

Medical students complete a “home-grown” exam of objective knowledge at the completion of their rotation and additionally complete a survey regarding their experiences in several core competency areas as specified by the ACGME.^16^ The medical student survey evaluates curriculum organization, patient care experience, bedside education, student perception of faculty and residents as educators, problem-based learning and improvement (PBLI), communication, professionalism, and use of systems-based practice on a five-point Likert scale. The student survey is administered to our students at the end of every clinical clerkship, and has been unmodified for several years, providing a readily available anonymous data set that the students were accustomed to provide multiple times a year. It is written and distributed by our Office of Student Affairs, and in addition to providing data for the ACGME, is used to generate anonymous feedback and highlight areas of excellence and deficiency for each clerkship.

### Study Protocol

Approval was obtained from this institution’s research review board prior to the initiation of any data collection. Between July 2010 and May 2012, medical student survey results, end-of-rotation test scores, and NEDOCS scores were collected and reviewed on an ongoing basis following the completion of each rotation. This period included 20 four-week rotation blocks and one eight-week rotation block for a total of 21 blocks. A total of 211 students rotated through the ED in this time period, with between five and 21 (in the eight-week block) students rotating in the department at one time.

### Measurements

First described in 2004, the NEDOCS score quantifies the level of ED crowding by measurement of several variables related to current ED patient load, admitted patient boarding time, and available hospital beds.^14^ The resulting score is divided into ranges of values denoting normal volume (NEDOCS<60), busy (61–100), crowded (101–140), dangerously crowded (141–180), and disaster-level crowding (>180). These results appear in Table 1. Data were obtained for this via review of records from the ED’s electronic medical record (EDIMS LLC; Parsippany, NJ). The NEDOCS score is calculated and recorded hourly at our institution, and the crowding numbers from 9 a.m. to 11 p.m. of each block were analyzed.

Student test scores had a maximum value of 100 points; student survey responses were scored on a five-point Likert scale, where one indicated strong disagreement with a given statement and five indicated strong agreement. The student survey appears in Appendix 1.

### Data Analysis

We calculated means for student survey results and end-of-rotation test score by rotation period. Weighted regression analyses determined the association between either average medical student test scores or average survey responses and percent crowding among the 21 blocks between the hours of 9 a.m. to 11 p.m. Weighting simultaneously accounted for heterogeneous variation in the block-level means due to differences between periods in the standard deviations (SDs) (in some cases the largest SD was 10 times as large as the smallest SD) and in the number of students (ranging from five to 21 for test scores and from four to 14 for the survey). The weights were set equal to the inverse of the squared standard errors of the block average for each particular response. Regression coefficients, p-values and r-squared values were calculated to assess the fit of the weighted regression models. We performed all data analysis using SAS 9.3 for Windows.

## RESULTS

During the period of the study, 211 students rotated through the ED in 20 four-week blocks and one eight-week block, and a total of 10,047 clinical exposure hours were recorded. Dividing the clinical hours via NEDOCS, 19% of the period recorded as crowded, in 3.4% of the study hours the ED was dangerously crowded; and in 0.7% of the study period, the ED was at disaster-level crowding. These results appear in Table 1. The means, SDs, standard errors and ranges of these for the student survey responses and test scores, based on the 21 blocks of students, are summarized in Table 2. The ranges of standard errors for the means indicate the need for weighted regression analysis. NEDOCS scores were most highly correlated with end-of-rotation test scores (p=0.0003) and student evaluations of communication, systems-based practice, and bedside education (p=0.0059, 0.023, and 0.016, respectively). In all cases, the association was negative, indicating an inverse correlation between crowding and positive survey responses/end-of-rotation test scores; i.e, the more crowded the ED was, the worse the survey responses and test scores were. However, associations with student evaluations of patient care, faculty/resident teaching, PBLI, and professionalism did not reach statistical significance.

Examination of the NEDOCS score distributions revealed a single outlier that was more than 10 points higher than the next highest block’s NEDOCS score. This had the potential to unduly influence the results of the regression analysis; thus, the analyses were repeated with this value excluded. With this value excluded, we recalculated the regression coefficients and p-values (i.e., based on 20 blocks of data). These results appear in Table 3. Similar associations were noted as in Table 2. Again, NEDOCS score demonstrated a negative correlation with test score and student evaluation of bedside education, communication, and systems-based practice.

Further sensitivity analysis (data not shown) examined the effect of NEDOCS score after accounting for month of the year; in this analysis, the effect of the NEDOCS score was largely eliminated. Only end-of-rotation test score remained significant (p=0.011) and only when including all 21 blocks (i.e., including the time period with the outlying NEDOCS score). As before, there was an inverse correlation between the end-of-rotation test score and NEDOCS score. P-values for all other parameters were non-significant (p≥0.34). On review of the exam scores from block to block, there is a small tendency towards higher scores at the beginning and the end of the year, with the lowest scores tending to cluster around the middle of the year.

## DISCUSSION

Relatively few studies have assessed the relationship between ED crowding and educational outcomes. No previous studies to date have examined quantitative markers of educational performance in this setting. This study demonstrated that higher rates of crowding as measured by the NEDOCS score did have a negative effect on certain aspects of the medical student educational experience; this result is at odds with prior studies showing no relationship between educational measures and ED crowding.^9^–^11^ Prior studies have focused predominantly on resident physicians, making this study unique in its focus.

Certain aspects of the medical student rotation evaluation did achieve a statistically significant correlation with the NEDOCS crowding metric, while others did not. One potential explanation is that those elements that do show a correlation may be those most likely to be affected by a crowded ED (in particular, communication and bedside education). Certain other aspects (e.g., professionalism and patient care) may be relatively unaffected as attending physicians view these as more critical elements to maintain regardless of crowding status.

Attending physicians may employ trade-offs that sacrifice certain aspects of the educational process—bedside teaching, exploring a student’s differential, or expanding upon teaching points for example—in favor of retaining strategies that maximize patient flow and direct patient care when the ED is crowded.^17^ A previous study of resident evaluation of attending physician teaching quality found little association between attending workload and quality of teaching; other factors (interpersonal skill, willingness to teach) had the greatest effect on perceived teaching quality.^18^ Supervision of EM residents is known to be adversely affected by ED crowding;^19^ a similar situation may apply to medical student education. There may even be a larger effect as attendings have less individually vested in a student doing a four-week rotation vs. a full EM residency, and EM residents should have more experience learning in the opportunistic, unscheduled, and possibly chaotic learning environment of a crowded department.

The findings demonstrated here conflict somewhat with those of Berger et al., who found no correlation between ED attending physician productivity as measured in relative value units (RVU) and medical student evaluation of their clinical teaching.^20^ This study did not address physician productivity per se, but it does raise the issue of whether teaching quality is the primary driver in the medical student performance outcomes. It may be the case that despite unchanged teaching quality by attending physicians, the retained knowledge by students may be lower in a crowded ED setting. If true, this argues that environmental effects of a crowded ED (e.g., noise, lack of workspace, frequent task-switching) may play a greater role in the negative effects on medical student experience than interaction with the attending physicians.

Regardless of the individual medical student rotation evaluation results, the end-of-rotation examination did show a negative correlation with ED crowding. The medical students rotating at the study facility receive mandatory weekly educational lectures as well as a suture workshop during their rotation. These didactic experiences are essentially the same for each block of students (the same PowerPoint presentations are given by different members of our attending faculty), making their ED experience the most variable part of the rotation itself and, presumably, the factor most likely to explain variation in their end-of-rotation test scores. Individual medical student motivations may play a role as well; this is discussed in more detail below.

Future investigations should include similar objective measures of student performance and may benefit from comparisons between multiple measurements of ED crowding (NEDOCS, EDWIN, etc.). Additionally, repeating this study with a cohort analysis of EM-applicant medical students versus non-EM applicant medical students would be of interest; this could better elicit the effect of student motivation on rotation experience A standardized National Board of Medical Examiners (NBME) subject examination has replaced the home-grown examination previously used at the study institution; the study could be repeated once sufficient data has been accumulated to allow for the opportunity to expand this analysis beyond the study institution and to standardize results between different institutions.

## LIMITATIONS

Our data indicate a possible confounding effect with month of the year on the association between the NEDOCS score and end-of-rotation exam score. Availability of only two years’ worth of data limits the power to analyze this effect fully and may lead to over-adjustment by month. However, some kind of temporal association is not unexpected, as the cohort of medical students rotating in the ED varies in its characteristics throughout the year. In the late summer and fall, medical students who plan to apply for EM residences complete their ED rotations. This likely represents a different group with distinct motivations from those who complete ED rotations later in the year, following the residency application period. The former group is likely more aggressive in attaining educational goals despite the potentially crowded state of the ED. Presumably, they may also have had exposure to an ED in the past, giving them some familiarity of how to function in a busy environment. Additionally the number of medical students in a block has significant variation–during the study period we combined what was previously the end of November through January blocks into one extended block and accepted more students into that block. Immediately after the change we reviewed both the student exam scores and their feedback from having a more spread-out schedule and found no differences from the other blocks and previous years. Based on this, we did not specifically exclude the data from that block, This explanation remains speculative, however, and requires further investigation.

Limitations of this study also include small and differing sample sizes within each group of medical students. As noted previously, there is a suggestion of a confounding effect due to month of the year, but more data would be needed to confidently estimate its effect. The study was limited to a single site and did not include the full 24 hours of ED crowding data (We analyzed only the periods of 9 a.m. to 11 p.m. as students do only one overnight shift during their rotation, and the NEDOCS score often falls dramatically during the overnight hours.) When we designed the study, we were aware of how seldom we were crowded on the overnight hours, and that combined with the facts that the students did few overnight shifts led us to exclude the overnight shift in the initial design of the study. In retrospect we probably should have left the overnight shift in the data set and used their exclusion/inclusion as another variable. We analyzed the medical student performance on the basis of their averaged group performance, rather than individual student results for each block in question. Additionally, the end-of-rotation examination used in this study was developed jointly by the clerkship directors and educational faculty within the study site and our sister institution; it is not a standardized examination, and it is not validated. Our site has begun using the NBME advanced subject exam in EM, but it was not yet available at the time of this study. Also, the medical student experience survey, which is administered by our medical school at the end of every clinical clerkship, is to the best of our knowledge also a home-grown and not validated survey. It was written or at least modified by members of the Office of Student Affairs to capture data for the ACGME and within institution use. It was selected as a marker of subjective student experience because the students, and through them the dean and department chairs, at our school use this survey as the primary method to obtain student feedback on their clinical clerkships. Individual medical student motivations also lie outside the ability of this study to detect, though a cohort analysis of EM-applicant medical students versus non-EM applicants could potentially isolate the effect of medical student motivation as a contributor to outcome.

## CONCLUSION

Our study sought to assess connections between objective measures of ED crowding and objective and subjective measures of medical student experience in the ED. A weak negative association was noted between end-of-rotation test scores and NEDOCS scores when considering the entire time period of this study and accounting for variability associated with month of the year. No subjective measure of rotation experience was correlated with the NEDOCS score when accounting for month of the year, which is in accordance with prior studies that have not suggested any effect of ED crowding on medical education.^9^,^10^ The limited association found in this study suggests that ED crowding has a negative effect on medical student education. These results can be applied practically now to schedule medical student rotations for periods in which crowding is expected to be lower, potentially leaving open the opportunity for more educational time.

## Supplementary Information



## Figures and Tables

**Table 1:** National Emergency Department Overcrowding (NEDOC) scale data for study period.

Block of study period	Average NEDOC score	Percent of time overcrowded	Percent of time dangerously overcrowded	Percent of time at disaster level
1	69.1	16.6%	7.5%	4.8%
2	58.2	8.3%	1.8%	0.9%
3	63.4	13.9%	3.6%	2.2%
4	61.5	12.5%	4.9%	2.0%
5	66.2	16.1%	4.7%	0.2%
6	64.7	15.2%	0.9%	0.0%
7	75.7	25.4%	9.8%	2.0%
8	68.7	18.8%	2.9%	0.7%
9	65.8	17.9%	1.6%	0.0%
10	67.2	16.1%	2.2%	0.0%
11	52.8	6.3%	0.0%	0.0%
12	58.7	8.3%	0.4%	0.0%
13	58.6	7.4%	3.6%	2.2%
14	76.8	27.7%	3.3%	0.0%
15	70.5	20.1%	1.1%	0.0%
16	76.1	28.6%	4.5%	0.7%
17	69.3	19.7%	3.2%	0.3%
18	70.7	21.9%	0.4%	0.0%
19	77.2	30.4%	2.5%	0.0%
20	74.8	25.9%	3.6%	0.4%
21	90.1	41.3%	9.2%	0.0%
Average	68.4	19%	3.4%	0.7%

**Table 2:** Summary statistics for National Emergency Department Overcrowding Scale (NEDOCS) scores and medical student outcomes along with regression coefficients, p-values, and r-squared values summarizing relationship between NEDOCS scores and the outcomes.

Variable	Mean (SD)	Range of averages	Range of SE*	Regression coefficient	p-value	R^2^ value
NEDOCS	68.40 (8.12)	52.8–90.1	--	--	--	--
Test score	83.78 (1.88)	80–86.77	1.04–3.34	−0.16	0.0003	50%
Organization	4.35 (0.24)	3.67–4.68	0.02–0.20	0.0077	0.065	17%
Patient care	3.91 (0.23)	3.34–4.31	0.17–0.80	0.0029	0.66	1%
Bedside education	3.84 (0.34)	3.14–4.54	0.02–0.24	−0.018	0.016	27%
Faculty/resident teaching	4.10 (0.25)	3.60–4.57	0.02–0.19	0.0061	0.20	9%
PBLI	4.04 (0.28)	3.34–4.57	0.00–0.18	0.0069	0.24	7%
Communication	4.19 (0.23)	3.77–4.69	0.02–0.35	−0.013	0.0059	34%
Professionalism	4.26 (0.22)	3.70–4.71	0.00–0.25	0.0055	0.43	4%
Systems-based practice	4.16 (0.23)	3.74–4.61	0.02–0.19	−0.011	0.023	24%

*SD,* standard deviation; *PBLI,* problem-based learning and improvement

* Range of standard errors (SE) of responses as calculated by block. The square of these values are used for weighting in the weighted regression analysis.

**Table 3:** Summary statistics for National Emergency Department Overcrowding Scale (NEDOCS) scores and medical student outcomes when data for single outlying block with NEDOCS >80 were excluded.

Variable	Regression coefficient	p-value	R^2^ value
Test score	−0.1400	0.0042	37%
Organization	0.0041	0.43	3%
Patient care	−0.0097	0.23	8%
Bedside education	−0.0260	0.0015	44%
Faculty/resident teaching	−0.0084	0.26	7%
PBLI	−0.0030	0.73	1%
Communication	−0.020	<0.0001	62%
Professionalism	0.0039	0.67	1%
Systems-based practice	−0.0140	0.0024	41%

*PBLI,* problem-based learning and improvement
