# Supplementary material for: How Does Emergency Department Crowding Affect Medical Student Test Scores and Clerkship Evaluations?
Source: West J Emerg Med. 2015 Nov 12;16(6):913–8. doi: 10.5811/westjem.2015.10.27242 (PMC4651593; doi:10.5811/westjem.2015.10.27242)
Supplement: Supplementary file 1 [file wjem-16-913-s001.pdf]

Appendix 1. Survey given to medical students upon completing their emergency medicine rotation at the study site.

Topics 1-8 were scored on a 1-5 scale, with 1 indicating strong disagreement and 5 indicating strong agreement.

1. Please provide you opinion about the organization of the clerkship.
    a. The orientation clearly outlined clerkship goals and objectives.
    b. The orientation established clerkship requirements, including the grading policy.
    c. I received formal mid-clerkship feedback.
    d. My required clinical encounters were formally documented by me and reviewed by a supervising attending or resident.
    e. The clerkship director was approachable.
    f. Overall, the clerkship was well organized.
    g. The material presented in core lectures was pertinent and helped me in the clerkship.
2. Please provide your opinion about your patient care experience on the clerkship.
    a. My involvement and responsibility in the care of patients was appropriate.
    b. There was sufficient opportunity to practice and improve my history and physical examination skills.
    c. The clerkship was effective in improving my clinical decision-making skills.
    d. The clerkship helped develop by patient management skills.
    e. The outpatient/ambulatory experience was educational and worthwhile.
3. Please provide your opinion about your educational experience on the clerkship.
    a. My performance on the clerkship was assessed against the learning objectives for the clerkship.
    b. A faculty member personally observed me taking a patient history during the clerkship.
    c. A faculty member personally observed me performing a major component of the physical examination during the clerkship.
    d. The clerkship expanded my knowledge of core topics, including disease pathophysiology.
    e. Instruction in clinical pharmacology was provided during this clerkship.
4. Please provide your opinion about the clinical instructors (faculty and/or residents) that you encountered on this clerkship.
    a. I received constructive feedback that was timely enough to be useful.
    b. Overall, they were interested in teaching and took the time to help me understand the material.
    c. At least one faculty member knew me well enough to evaluate my performance.
    d. I was evaluated systematically and fairly.
    e. Overall, I was well supervised.
5. Practice-based learning and improvement. Please comment on the following:
    a. I was able to get help with my learning when needed.

    b. I was exposed to current medical literature on this clerkship.
    c. I had an opportunity to review and evaluate information from recent clinical trials as it pertained to clinical decision-making.
    d. Data with regards to diagnostic screening and risk management were discussed with me.
    e. Teamwork was valued on this clerkship.
6. Interpersonal and communication skills. Please provide your opinion about the following:
    a. Developing a good relationship with my patient was valued by my team.
    b. I had opportunities to improve my communication skills during this clerkship.
    c. I encountered culturally diverse patients on this clerkship.
    d. I observed informed consent being obtained from a patient.
    e. I observed the effective delivery of bad or distressing news.
    f. I observed a clinical instructor deal effectively with a difficult (e.g. angry or manipulative) patient.
7. Professionalism. Please provide your opinion about the following:
    a. Honesty was valued by the faculty and residents.
    b. Patient confidentiality was a priority for my team.
    c. Patient comfort and privacy was respected whenever possible.
    d. Faculty demonstrated sensitivity and respect towards patients.
    e. Residents demonstrated sensitivity and respect towards patients.
    f. Faculty, Residents, and Nurses treated students with courtesy and respect.
    g. Professional behavior by faculty, residents, or nurses made for a conducive learning environment.
8. Systems-based practice. Please provide your opinion about the following:
    a. Cost-effective decision making was discussed with me.
    b. I provided care for uninsured patients.
    c. I was made aware of the challenges in access to health care for the uninsured.
    d. We used consultation resources (discharge planning, social services, etc.) to ensure patients received the care they needed.
    e. I was made aware of common medical errors.
    f. Patient safety was a priority for our service.
    g. We discussed complications or bad outcomes to determine where we could improve the delivery of care.
9. Please comment on the following:
    a. What do you consider to be the strengths of this clerkship?
    b. What do you consider to be the weaknesses of this clerkship?
    c. What changes would you recommend to address these weaknesses?
